# High Lithium Content and Site Disorder in the Transition Metal Oxide Argyrodites Li_7_TiO_5_X (X = Cl^–^, Br^–^)

**DOI:** 10.1021/acs.chemmater.6c00177

**Published:** 2026-06-22

**Authors:** Alexandra Morscher, Lucia Corti, Samuel L. Goodwin, Andrés Acín-Lalanza, Matthew A. Wright, T. Wesley Surta, Ruiyong Chen, Matthew S. Dyer, Frédéric Blanc, Luke M. Daniels, John B. Claridge, Matthew J. Rosseinsky

**Affiliations:** a Department of Chemistry, 4591 University of Liverpool, Crown Street, L69 7ZD Liverpool, U.K.; b Leverhulme Research Centre for Functional Materials Design, Materials Innovation Factory, University of Liverpool, 51 Oxford Street, Liverpool L7 3NY, U.K.; c Stephenson Institute for Renewable Energy, University of Liverpool, Peach Street, L69 7ZF Liverpool, U.K.

## Abstract

Sulfide lithium argyrodites are a key materials family that are studied as solid electrolytes in commercial all-solid-state batteries (ASSBs), while their oxide analogues remain relatively unexplored. This study presents the discovery of Li_7_TiO_5_X (X = Cl^–^, Br^–^), the first lithium argyrodite materials in which a transition metal is used as the framework-forming cation, expanding the chemical space that is accessible for oxide argyrodites. Incorporation of Ti^4+^ enables the lithium content to be maximized to 7 Li^+^ per formula unit. Interestingly, even with the high lithium content, Li_7_TiO_5_Cl retains a Li^+^ site disordered cubic *F*4̅3*m* structure at room temperature with Li^+^ occupancy of the T5, T5a, and T3 positions, and exhibits an ionic conductivity of 2.2(2) × 10^–6^ S cm^–1^ with the lowest reported activation energy (0.36(2) eV) for bulk Li^+^ ion transport in an oxide argyrodite. Conversely, Li_7_TiO_5_Br adopts the same *F*4̅3*m* symmetry at room temperature but with an ordered arrangement of Li^+^ positions via full occupancy of the T5a and T3 positions, and thus has an ionic conductivity that is 3 orders of magnitude lower (∼10^–9^ S cm^–1^) and a much higher activation energy (0.58(2) eV) than Li_7_TiO_5_Cl. Order–disorder behavior is observed below 250 K in Li_7_TiO_5_Cl, where a Li^+^ site ordering pattern is observed that is distinct from Li_7_TiO_5_Br and all sulfide argyrodites, yielding a tetragonal symmetry (*I*4̅) for only the second time to date in the argyrodite structure type. This unique order–disorder behavior, alongside the ability to incorporate transition metal cations within this material family emphasizes the potential to access much greater structural diversity via the expansive chemical space that is available for exploration in oxide argyrodites.

## Graphical abstract

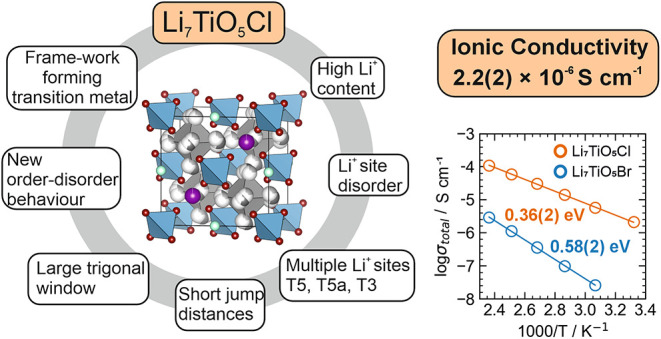

## 1 Introduction

Lithium-ion conductors are used in a range of electrochemical technologies, including in thin-film batteries,^1,2^ as protective coatings in lithium-ion batteries (LIBs),^3,4^ and as solid electrolytes (SEs) in all-solid-state batteries (ASSBs).^5,6^ For example, lithium phosphorus oxynitride (LiPON) is widely employed in commercial thin-film batteries, exhibiting room-temperature ionic conductivities of approximately 10^–6^ S cm^–1^ and good electrochemical stability. Several oxide SEs are used as protective coatings to stabilize electrode–electrolyte interfaces, including Li_2_SiO_3_,^7^ Li_4_Ti_5_O_12_,^8^ LiTaO_3_,^9^ and LiNbO_3_ ^10^ with ionic conductivities ranging from 10^–5^ S cm^–1^ (amorphous LiNbO_3_)^10^ to 10^–8^ S cm^–1^ (Li_5_Ti_5_O_12_).^10^ Lithium-conducting SEs with room-temperature conductivities that surpass those of organic liquid electrolytes (>10^–3^ S cm^–1^), such as lithium argyrodites, *e.g.*, Li_6.6_Si_0.6_Sb_0.4_S_5_I (2.4 × 10^–2^ S cm^–1^),^11^ can be used to develop ASSBs, which offer the prospect of enhanced safety alongside increased power and energy densities.^12,13^ Almost all reported lithium argyrodites utilize sulfide as the main anion, benefiting from the polarizability of the S^2–^ anion that enhances Li^+^ ion mobility.^14^

Argyrodites, characterized by the general formula A_(12–*n*–*y*)/*m*_^*m*+^M_6–*y*_^*n*+^Ch_6–*y*_^2–^X_*y*_^–^ (*A* = Ag^+^, Cu^+^, Cd^2+^, Li^+^, etc.; M = tetrahedral forming cations; *Ch* = chalcogenide; X = halides), exist in a variety of compositions. Most argyrodites are based on nonmetallic cations (*M*), such as P^5+^, Si^4+^, and Ge^4+^, that form tetrahedral units with *Ch* anions (*Ch* = O^2–^, S^2–^, Se^2–^, Te^2–^) (Figure S1).^15^ A limited number of argyrodites which contain transition metal cations have been explored, e.g., Ag_7_TaS_6_, Ag_7_NbS_6_,^16^ and Ag_8_TiS_6_,^17^ all with *F*4̅3*m* symmetry. Notably, no transition-metal-containing lithium argyrodites have been reported to date. This is likely due to the tendency of transition-metal sites to undergo redox reactions, which can generate electronically conductive interfacial layers, increase interfacial impedance, and accelerate degradation, rendering the materials unsuitable as separators in ASSBs.^18^ However, replacing oxygen with sulfur may help to suppress redox activity. Significant progress has been made recently with the discovery of Li*M*OCl_4_ (M = Nb^5+^, Ta^5+^)^19,20^ and Li_3_Ta_3_O_4_Cl_10_ ^21^ that incorporate transition metals alongside oxide and halide anions while exhibiting greater resistance to the conditions experienced within electrochemical cells. Transition metals feature in other oxide structure types that have been studied as solid electrolytes, including the highly conducting NaSICON-type Li_1+*x*_Al_*x*_Ti_2–*x*_(PO_4_)_3_,^22^ perovskite-type Li_3*x*_La_2/3‑*x*_□_1/3–2*x*_TiO_3_ (LLTO),^23^ and Li_5_La_3_ *M*_2_O_12_ (M = Nb^5+^, Ta^5+^) garnets,^24^ but have not yet featured in oxide argyrodite solid electrolyte chemistry. Argyrodites, which combine O^2–^ and halide X^–^ anions, are underexplored as potential solid-state electrolytes relative to sulfide argyrodites and may offer enhanced electrochemical stability. The first reported oxide argyrodite Li_6_PO_5_X (X = Cl^–^, Br^–^) exhibited low ionic conductivities of ∼10^–9^ S cm^–1^.^25^ Recent optimization of composition through silicon substitution in Li_6+*x*_P_1–*x*_Si_*x*_O_5_Cl led to a 1000-fold improvement in ionic conductivity to 1.82(1) × 10^–6^ S cm^–1^, demonstrating that substantial improvements to ionic conductivity are possible for oxide argyrodite materials.^26^ Typically, a lithium content greater than 6 Li^+^ per formula unit leads to high Li^+^ ion diffusion, as demonstrated in many argyrodite solid solutions, e.g., Li_6+*x*_P_1–*x*_M_*x*_S_5_I (M = Si^4+^ and Sn^4+^)^27^; however, argyrodites with a lithium content of 7 Li^+^ per formula unit are comparatively rare, for which Li^+^ site ordering is commonplace, negatively impacting the ionic conductivity.^26,28^

Our aim is to expand the chemical space accessible to argyrodites beyond the small number of typical framework-forming tetrahedral cations that dominate sulfide argyrodite chemistry (i.e., P^5+^, Si^4+^, Ge^4+^, As^5+^, Sb^5+^, Sn^4+^). Thus, we target the compositions of Li_7_TiO_5_X (X = Cl^–^, Br^–^) through solid-state reaction utilizing the Ti^4+^ valence to maximize the Li^+^ content. Both Li_7_TiO_5_Cl and Li_7_TiO_5_Br adopt a cubic argyrodite structure (*F*4̅3*m*) at room temperature. Li_7_TiO_5_Br exhibits an ordered structure with Li^+^ ions fully occupying T5a and T3 sites, while Li_7_TiO_5_Cl displays Li^+^ site disorder with partial occupancy of T5 and T5a and full occupancy of T3 sites at room temperature. This difference in Li^+^ site distribution has a profound impact on the transport properties of the two materials, with an increase in ionic conductivity of 3 orders of magnitude from Li_7_TiO_5_Br to Li_7_TiO_5_Cl, highlighting the importance of disorder to achieve increased ionic conductivities. Furthermore, Li_7_TiO_5_Cl undergoes a phase transition to a Li^+^ site-ordered structure with tetragonal *I*4̅ symmetry below 250 K, a symmetry previously observed only once for all argyrodites. The ability to incorporate transition metal cations into oxide argyrodites emphasizes the extensive chemical space that is available for exploration and subsequent optimization in the underexplored field of oxide argyrodites.

## 2 Experimental Section

### 2.1 Synthesis

#### 2.1.1 Materials

TiO_2_ (anatase, nanoparticles, >99.0%), LiBr (>99.0%), and LiCl (>99.0%) were purchased from Sigma-Aldrich. Li_2_O (greater than 99.0%) was purchased from Alfa Aesar.

#### 2.1.2 Synthesis of Li_7_TiO_5_X (X = Cl^–^, Br^–^)

Li_2_O, TiO_2_, LiBr, and LiCl were dried under dynamic vacuum (<10^–4^ mbar) at 473 K (LiCl, LiBr), 773 K (TiO_2_), and 1223 K (Li_2_O) overnight before placing them inside an Ar-filled drybox (O_2_ < 0.1 ppm, H_2_O < 0.1 ppm). Drying the starting materials was critical to avoid the formation of antiperovskite Li_2_OHX (X = Cl^–^, Br^–^) phases and other impurity phases; thus, all reagents and samples were handled under dry inert atmospheres.

Li_2_O, TiO_2_, and LiCl or LiBr were mixed in the stoichiometric ratios using ball milling under an Ar atmosphere. The precursors were ball-milled in 1 g batches for a total time of 6 h (12 intervals of 10 min on followed by 20 min off) in 45 mL zirconia jars using seven zirconia balls (diameter: 10 mm) at a rate of 300 rpm. The resulting powder was then pressed into 5 mm diameter pellets using 300 MPa pressure. The pellets were placed into alumina crucibles, which were inserted into quartz tubes sealed with Swagelok end-caps through which dried argon gas was flown at a rate of 50 mL min^–1^. The samples were heated to 898 K for Li_7_TiO_5_Cl and 823 K for Li_7_TiO_5_Br at a ramp rate of 5 K min^–1^, held at the reaction temperature for 10 h, and cooled at a rate of 5 K min^–1^. The quartz tubes were closed airtight using the Swagelok end-caps and returned to the Ar-filled glovebox, where they were opened, and powders were ground in an agate mortar for further characterization.

### 2.2 Powder X-ray Diffraction (PXRD)

Routine assessment of sample purity was carried out using a Bruker D8 Discover diffractometer with monochromatic Cu radiation (Kα_1_, λ = 1.54056 Å) in Debye–Scherrer transmission geometry with sample powders loaded into 0.7 mm or 0.3 mm diameter borosilicate glass capillaries for Li_7_TiO_5_Cl and Li_7_TiO_5_Br, respectively.

Synchrotron X-ray diffraction (SXRD) was performed at Diamond Light Source, U.K., on high-resolution beamline I11 ^29^ for all samples. The data were recorded in transmission mode using a position sensitive detector (PSD, [0° < 2θ < 100°], λ = 0.82660 Å). The experiments were carried out at room temperature and at 100 K on samples introduced into 1 mm (Li_7_TiO_5_Cl) and 0.3 mm (Li_7_TiO_5_Br) diameter borosilicate glass capillaries.

Synchrotron variable-temperature X-ray diffraction (VT-XRD) experiments were performed on powder samples of Li_7_TiO_5_Cl that were introduced into 1 mm diameter silica capillaries in the temperature range of 100–298 K in 25 K steps on heating. To identify the phases present, databases (ICSD,^30^ PCD) were searched for known materials containing Li, Ti, O, Cl, and Br alongside possible contaminants originating from the synthesis procedure, such as Al, Zr, C, and H.

Rietveld refinements were carried out using TOPAS Academic.^31^ Initially, Pawley fits were performed on SXRD data, refining the lattice parameters and the background terms using a Chebyshev function with 12 parameters. The parameters refined from final Pawley fits were then used as starting points for Rietveld refinements, where the following parameters were refined: (1) Scale factor, (2) atomic coordinates, (3) isotropic displacement parameters, and (4) atomic occupancies: the occupancies of Ti^4+^ were set to unity, while Cl^–^, Br^–^, and O^2–^ were refined initially. Since all occupancies were refined to values close to unity, they were set to 1 in the refinement. Additional Li^+^ sites were identified in the Fourier difference map (FDM) through the presence of positive electron density, introduced into the model and refined (atomic coordinates, atomic occupancies, isotropic displacement parameters). The overall Li^+^ content for each composition was fixed to charge-balance the refined anion content.

### 2.3 Alternating Current (AC) Impedance Spectroscopy and Direct Current (DC) Polarization

Pellets of Li_7_TiO_5_X (X = Cl^–^, Br^–^) were prepared through reactive sintering by uniaxially pressing ∼30 mg of starting material in a 5 mm cylindrical steel die at a pressure of 300 MPa, followed by annealing under argon flow at the respective reaction conditions. Using this method, relative pellet densities up to 81% were achieved.

AC impedance measurements were conducted using an impedance analyzer (Keysight impedance analyzer E4990A). A sputtered gold coating of ∼300 nm thickness was used as the ion-blocking electrode. Sputtering was achieved in a glovebox using a Q150R sputter coater at a pressure of 10^–1^ mBar and a sputtering current of 40 mA. Temperature-dependent conductivity measurements were performed under a dry argon atmosphere in a frequency range of 2 MHz to 20 Hz (with an amplitude of 100 mV). Measurements were performed at room temperature and in the temperature range 323–573 K in 25 K steps. The ZView2 program was used to fit the impedance spectra with an equivalent circuit.

DC polarization data were collected on Au|Li_7_TiO_5_X|Au (X = Cl^–^, Br^–^) symmetric cells at 298 K. Constant voltages of 0.1–1 V were applied for ∼2 h, and the current variation with time was recorded. The DC polarization curves were recorded until a steady current was obtained at any applied voltage.

For low-temperature impedance measurements, pellets were mounted and sealed in a spring-loaded sample jig inside the glovebox, which was then placed into a Delta 9023 environmental test chamber. Argon gas was passed from the inner tube to the outer tube to protect samples from moisture/air. Data points were collected in the temperature range 303–233 K in 10 K steps with an equilibration time of 30 min for each step.

### 2.4 Compositional Analysis

The Li and Ti content was determined through inductively coupled plasma optical emission spectroscopy (ICP-OES). 10 mg of Li_7_TiO_5_X (X = Cl^–^, Br^–^) was dissolved in 100 mL of 1 M nitric acid. Data were collected using an Agilent Technologies 5110 ICP-OES instrument, and three measurements were collected per sample, and the average was used as the reported value. Calibrations were performed using appropriate elemental standards. The Cl and Br contents were measured via ion chromatography (IC) using a Metrohm 881 compact IC Pro with an 858 professional sample processor. 5 mg of Li_7_TiO_5_X (X = Cl^–^, Br^–^) was dissolved in 50 mL of ultrapure distilled water and measured in triplicate. Solutions were diluted by a factor of 10 for measurement, and data were corrected using appropriate lithium halide salts as standards.

### 2.5 Nuclear Magnetic Resonance (NMR) Spectroscopy

All NMR experiments were performed under static conditions on a 9.4 T Bruker Avance III HD spectrometer. Samples were flame-sealed in a 3.2 mm borosilicate glass insert placed inside a 4 mm ZrO_2_ rotor. A 4 mm high-temperature HX solid-state NMR probe was used to acquire spectra above room temperature, while experiments below room temperature were recorded with a 4 mm HXY solid-state NMR probe in double resonance mode. All ^7^Li solid-state NMR spectra were recorded at a Larmor frequency ν_0_ of 156 MHz and are referenced to the ^7^Li chemical shift of LiCl in D_2_O at 0 ppm. Pulse-acquire spectra were recorded using optimized 90° pulses at a radio frequency field amplitude of 83 kHz and recycle delays longer than five times the ^7^Li spin–lattice relaxation time constant in the laboratory frame, T_1_, determined from ^7^Li saturation recovery experiments. Temperature calibrations of the probes were performed with Pb­(NO_3_)_2_ as ^207^Pb NMR^32^ chemical shift thermometers (below room temperature and above room temperature up to 400 K) and with the CuI and CuBr phase transitions using ^63^Cu NMR.^33,34^ The largest errors associated with this method arise from temperature gradients in the sample, which were calculated using the isotropic peak line broadening and range from 5 to 20 K.

The fitting of Boltzmann sigmoid functions to the Δν­(*T*) vs *T* line narrowing curves enables access to the full width at half-maximum of the completely narrowed ^7^Li central transition in the fast motional regime (Δν_∞_), the full width at half-maximum of the ^7^Li central transition governed by ^7^Li–^7^Li homonuclear dipole interactions in the rigid lattice regime at temperatures below *T*_onset_ (Δν_R_), and the temperature corresponding to the inflection point of the Δν­(*T*) vs *T* line narrowing curve (*T*_inflection_). From this, it is possible to obtain mean Li-ion jump rates based on τ^–1^ = 2πΔν_R_.

### 2.6 Normal Mode Calculations

The normal modes of Li_7_TiO_5_Cl were calculated in the conventional cubic cell of the *F*4̅3*m* structure using periodic density functional theory (DFT) as implemented in VASP.^35^ Calculations were performed using the PBEsol functional,^36^ a plane-wave cutoff energy of 700 eV, and a 5 × 5 × 5 *k*-point grid. The projector augmented-wave method was used to treat core electrons.^37^ The geometry of the *F*4̅3*m* structure was initially optimized until all forces fell below 0.001 eV Å^–1^, and then normal modes were calculated using finite differences with a displacement of 0.01 Å. Following the identification of unstable modes with imaginary frequencies, new structures were generated by perturbing the *F*4̅3*m* structure along the direction of the unstable modes. These new structures were then optimized using the same parameters as those given above.

### 2.7 Diffuse Reflectance Measurements

Diffuse reflectance data of Li_7_TiO_5_X (X = Cl, Br) powders were measured using an Agilent Cary 5000 instrument between 200 and 2500 nm with a step size of 1 nm. Calibration to 100 and 0% reflectance was performed prior to measurement using a poly­(tetrafluoroethylene) (PTFE) standard and a light trap, respectively. Samples were loaded into holders inside a glovebox (O_2_ < 0.1 ppm, H_2_O < 0.1 ppm), and the threads of the holders were wrapped in PTFE tape to avoid atmospheric exposure of the material during measurement. The band gap was determined from a Tauc plot using a method described by Makuła et al.^38^

### 2.8 Bond Valence Sum Energy (BVSE) Calculations

The BVSE calculations were performed for Li_7_TiO_5_Cl −300 K and Li_7_TiO_5_Cl −100 K of Li-argyrodite using *softBV*(v1.3.1).^39–41^ The crystallographic information file (CIF), obtained through the Rietveld refinement, was assessed in *softBV*, and the global instability index (GII) was found to be no larger than 0.18 in both refined crystal structures; hence, the validity of the CIFs was confirmed. The input file (.cube) was generated in *softBV*, and the BVSE landscape was plotted in VESTA^42^ to visualize the Li-ion migration pathways.

## 3 Results and Discussion

### 3.1 Synthesis of Li_7_TiO_5_X (X = Cl^–^, Br^–^)

Syntheses of Li_7_TiO_5_X (X = Cl^–^, Br^–^) were attempted at several temperatures (723–923 K) and reaction times (3–24 h). Reactions executed at 898 and 823 K for 10 h under flowing Ar (50 mL min^–1^) resulted in the formation of phase-pure cubic argyrodite powders for Li_7_TiO_5_Cl and Li_7_TiO_5_Br, respectively. Syntheses for Li_7_TiO_5_Cl undertaken at 823 K resulted in samples of reduced purity (Figure S2a,b). The reflections observed in XRD patterns of both materials were consistent with the systematic absences characteristic of the *F*4̅3*m* space group often adopted by cubic argyrodites (*hkl*: *h* + *k*,*h* + *l*,*k* + *l* = 2*n*, 0*kl*: *k,l* = 2*n*, *hhl*: *h* + *l* = 2*n*, *h*00: *h* = 2*n*) (Figure 1a). The observed reflections are shifted to lower Q for Li_7_TiO_5_Br due to the increased ionic radii (r_Cl_: 1.81 Å vs r_Br_: 1.96 Å),^43^ with an associated increase in unit cell parameter from *a* = 8.527050(9) Å to *a* = 8.576847(11) Å, respectively. (Figure 1b). Additionally, differences in relative reflection intensities are observed between Li_7_TiO_5_Cl and Li_7_TiO_5_Br, most notably for the (11–1) reflection at ∼1.3 Å^–1^.

**Figure 1:**
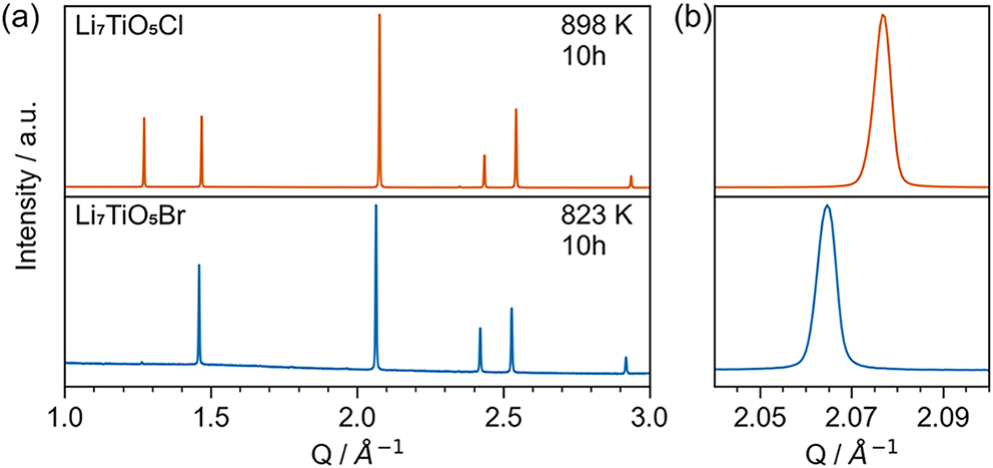
(a) Room-temperature XRD patterns for Li_7_TiO_5_Cl (orange) and Li_7_TiO_5_Br (blue) powder samples can be indexed to cubic *F*4̅3*m* symmetry typical of argyrodite materials. (b) Reflections shift to lower Q as the larger Br^–^ anion (blue) is substituted in place of Cl^–^ (orange).

### 3.2 Structure Determination

#### 3.2.1 Li_7_TiO_5_X: Room-Temperature Structure Determination

The crystal structures of Li_6_PO_5_X (X = Cl^–^, Br^–^),^25^ which adopt *F*4̅3*m* symmetry, were used as starting points for Rietveld refinements of Li_7_TiO_5_X (X = Cl^–^, Br^–^) by replacing the P^5+^ for Ti^4+^ and placing the one additional Li^+^ onto the tetrahedral T3 (4*d*) site, alongside the fully occupied trigonal bipyramidal T5a (24*g*) site. The total Li^+^ content in each model was set to charge-balance the refined anion content.

For Li_7_TiO_5_Br, refinement of the positions and displacement parameters of the Br^–^, O^2–^, Ti^4+^, and Li^+^ sites as well as the occupancies of the Li^+^ sites gave an *R*_wp_ = 1.20%, GOF = 4.10, and a good visual fit to the experimental data (Figure 2a, and Table 1). The isotropic displacement value of the T5a positions is comparatively high at 2.27(6) Å^2^, which may be indicative of local displacements of the Li^+^ ions potentially toward the T5 position. However, inclusion and refinement of the T5 site as well as other additional Li^+^ sites (T4, T2) in the model for Li_7_TiO_5_Br led to negligible occupancies, with no evidence for electron density on those sites in the Fourier difference map (FDM). A composition of Li_6.96(5)_Ti_1.00(5)_O_5_Br_1.09(1)_ is measured via analytical methods for Li_7_TiO_5_Br (Table S1).

**Figure 2:**
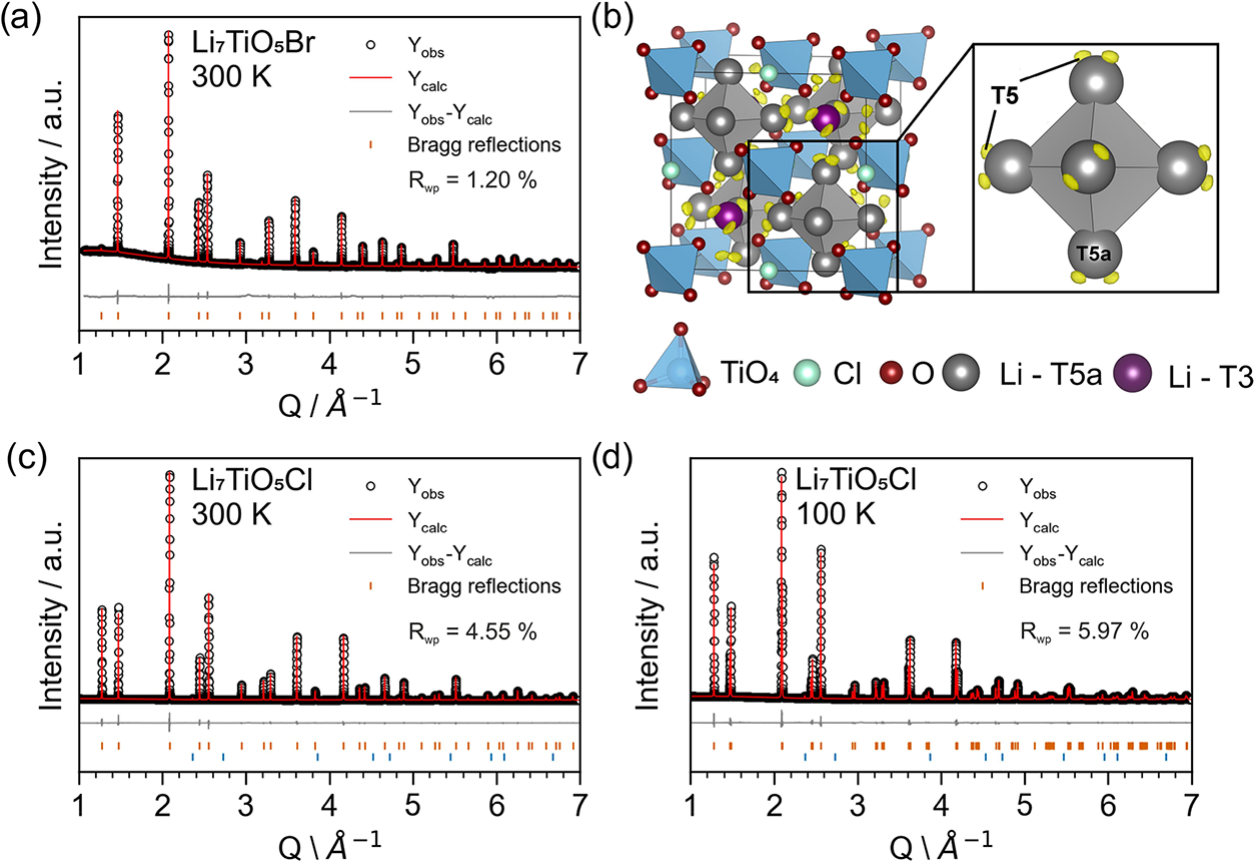
(a) Rietveld refinement of Li_7_TiO_5_Br against high-resolution synchrotron XRD data at 300 K. (b) Fourier difference map obtained from Rietveld refinement against 300 K PXRD data of Li_7_TiO_5_Cl with Li^+^ modeled on the T5a and T3 positions, highlighting additional electron density (yellow) observed on the T5 position. (c) Rietveld refinement of Li_7_TiO_5_Cl against high-resolution synchrotron XRD data at 300 K. (d) Rietveld refinement of Li_7_TiO_5_Cl against high-resolution synchrotron XRD data at 100 K. *Y*_obs_ (black circles), *Y*_calc_ (red line), *Y*_obs_–*Y*_calc_ (gray line), and Bragg reflections (orange tick marks for Li_7_TiO_5_X (X = Cl^–^, Br^–^) and blue tick marks for 0.99 wt % Li_2_O impurity).

For Li_7_TiO_5_Cl, refinement of the Cl^–^, O^2–^, and Ti^4+^ positions and displacement parameters gave a reasonable visual fit to the experimental data but with clear intensity discrepancies and an *R*_wp_ of 9.25% (Figure S2a). In contrast to the model refined for Li_7_TiO_5_Br, the displacement parameter for the Li^+^ T5a site (24*g*) in Li_7_TiO_5_Cl was refined to a large value of 5.31 Å^2^ compared to 0.87 Å^2^ for the T3 site. Additional electron density centered around the T5 site in proximity to the T5a site was visible upon inspection of the FDM (Figure 2b). Lithium was introduced onto the T5 site with the combined maximum occupancies of the T5 and T5a sites set to unity. The refined site occupancy factor (*s.o.f.)* for T5a and T5 sites were 0.6013(14) and 0.199(7), respectively, and both sites then displayed satisfactory displacement parameters (1.881(8) Å^2^ and 0.43(12) Å^2^, respectively). The final refinement gave an *R*_wp_ of 4.55% and GOF of 3.64 (Table 1) and a good visual fit (Figure 2c) to the data. Analytical methods return a composition of Li_6.86(7)_Ti_1.00(3)_O_5_Cl_0.968(9)_ for Li_7_TiO_5_Cl (Table S1). It is noted that these refinements against high-resolution synchrotron X-ray data extract the maximum amount of information available from the data, and that collection of neutron diffraction data could benefit the further study of Li^+^ ion migration in such disordered materials.

#### 3.2.2 Order–Disorder Behavior at Low Temperature in Li_7_TiO_5_Cl

Li_7_TiO_5_Br retains the same cubic *F*4̅3*m* symmetry and ordered distribution of Li^+^ sites (T5a and T3 sites) at 100 K as is seen at 300 K (Figure S2b). In contrast, the data collected at 100 K for Li_7_TiO_5_Cl revealed splitting of some reflections, indicating a transition to a lower structural symmetry (Figure 3a). The reflections observed in the 100 K data could be indexed to the tetragonal *I*4̅ space group (*hkl*: *h + k + l* = 2*n*, *hk*0: *h + k* = 2, 0*kl*: *k + l* = 2*n*, *hhl*: *l* = 2*n*, 00*l*: *l* = 2*n, h*00: *h =* 2*n*), a possible subgroup of *F*4̅3*m*, with lattice parameters of *a* = 5.992190(14) Å and *c* = 8.550351(3) Å. The resulting *I*4̅ unit cell is related to the high-symmetry *F*4̅3*m* cell via: $a_{t}=\sqrt{2}\times \frac{a_{c}}{2},c_{t}=a_{c}$ (where *a*_*t*_, *c*_*t*_, and *a*_*c*_ denote the lattice parameters for the tetragonal (*t*) and cubic cell (*c*)). Variable-temperature XRD data collected from 100 to 300 K in 25 K steps revealed the onset of reflection splitting below 250 K (Figure 3b). The observed reflection splitting is most prominent at 100 K with a *c*_*t*_/*a*_*c*_ lattice parameter ratio of 1.009. Convergence of the *a* and *c* parameters (at *c*/*a* = 1) is observed as the temperature is increased to 250 K (Figure 3c), as well as a discontinuity in the change in unit cell volume (Figure 3d).

**Figure 3:**
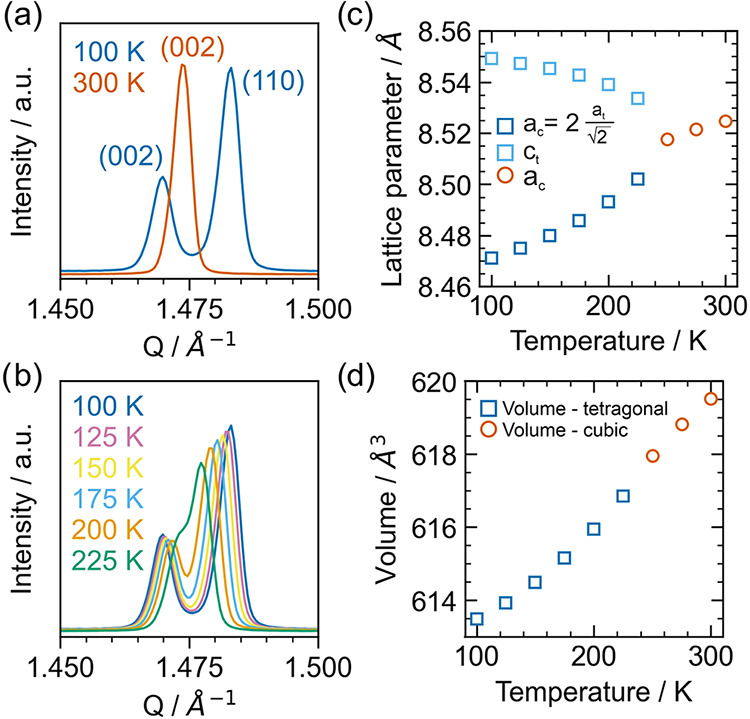
(a, b) Reflection splitting consistent with *I*4̅ symmetry is observed for Li_7_TiO_5_Cl below room temperature with a transition between *F*4̅3m and *I*4̅ at 250 K. (a) The (002) reflection at 300 K (orange) is split into (002) and (110) reflection at 100 K (blue), with (b) the convergence of the (002) and (110) reflections in the temperature range 100–225 K. (c) Variation in lattice parameters as a function of temperature for Li_7_TiO_5_Cl, lattice parameter *a* in the tetragonal unit cell is plotted using the pseudo-cubic unit cell with the conversion: $a_{c}=2\times \frac{a_{t}}{\sqrt{2}}$ and (d) variation in unit cell volume as a function of temperature for Li_7_TiO_5_Cl.

The starting model for refinement of the Li_7_TiO_5_Cl *I*4̅ structure at 100 K was obtained from normal mode calculations, which suggested a set of stable lower-symmetry structures arising from Li^+^ displacement from the high-symmetry positions within the room-temperature *F*4̅3*m* structure (Tables S2–S3). The most energetically favorable among these was a tetragonal structure with *I*4̅ symmetry, in excellent agreement with the observations from experiment. The Cl^–^, O^2–^, and Ti^4+^ positions and displacement parameters as well as the Cl^–^ occupancy were refined prior to careful assessment of the Li^+^ sites analogous to the room-temperature structures, with the overall Li^+^ content set to charge-balance the refined anion content. Refinement of Li^+^ positions (T5a, T5), occupancies (T5a, T5, T3) and displacement parameters (T5a, T5, T3) produced the final Rietveld fit with *R*_wp_ = 5.97% and GOF = 4.64 (Figure 2d and Table 1).

**Table 1:** Atomic Positions, Isotropic Atomic Displacement Parameters (*B*_iso_, Å^2^), and Site Occupancy Factors for Li_7_TiO_5_Cl and Li_7_TiO_5_Br Obtained from Rietveld Refinement against X-ray Powder Diffraction Data at 300 and 100 K

site	Wyckoff position	*x*	*y*	*z*	*s.o.f.*	*B*_iso_ (Å^2^)
Li_7_TiO_5_Br-300 K-*F*4̅3*m*–a = 8.576847(11) Å-*R*_wp_ = 1.20%						
Ti1	4*a*	0	0	0	1.0000	0.34(2)
Br1	4*b*	0.5	0.5	0.5	1.0000	1.051(18)
O1	16*e*	0.88106(9)	0.88106(9)	0.88106(9)	1.0000	0.91(3)
O2	4*c*	0.25	0.25	0.25	1.0000	1.02(5)
Li1 (T5a)	24*g*	0.4748(4)	0.25	0.25	1.0000	2.27(6)
Li2 (T3)	4*d*	0.75	0.75	0.75	1.0000	0.17(10)
Li_7_TiO_5_Br-100 K-*F*4̅3*m* −a = 8.562056(11) Å-*R*_wp_ = 1.16%						
Ti1	4*a*	0	0	0	1.0000	0.293(15)
Br1	4*b*	0.5	0.5	0.5	1.0000	0.20(1)
O1	16*e*	0.88043(8)	0.88043(8)	0.88043(8)	1.0000	0.55(3)
O2	4*c*	0.25	0.25	0.25	1.0000	0.19(4)
Li1 (T5a)	24*g*	0.4770(4)	0.25	0.25	1.0000	1.31(8)
Li2 (T3)	4*d*	0.75	0.75	0.75	1.0000	0.20(18)
Li_7_TiO_5_Cl-300 K-*F*4̅3*m* −a = 8.527040(9) Å-*R*_wp_ = 4.55%						
Ti1	4*a*	0	0	0	1.0000	0.264(4)
Cl1	4*b*	0.5	0.5	0.5	1.0000	1.592(7)
O1	16*e*	0.87933(4)	0.87933(4)	0.87933(4)	1.0000	0.782(11)
O2	4*c*	0.25	0.25	0.25	1.0000	0.685(10)
Li1 (T5a)	24*g*	0.4753(4)	0.25	0.25	0.6013(14)	1.881(8)
Li2 (T5)	48*h*	0.71141(2)	0.71141(2)	0.4658(5)	0.199(7)	0.43(12)
Li3 (T3)	4*d*	0.75	0.75	0.75	1.0000	0.56(5)
Li_7_TiO_5_Cl-100 K-*I*4̅ −a = 5.992190(14) Å; *c* = 8.550351(3)Å-*R*_wp_ = 5.97%						
Cl1	2*a*	0	0	0	1.0000	0.655(9)
Ti1	2*b*	0	0	0.5	1.0000	0.203(6)
O1	8*g*	0.5136(3)	0.25874(13)	0.12209(9)	1.0000	0.381(12)
O2	2*c*	0	0.5	0.25	1.0000	0.224(16)
Li1 (T5a)	4*f*	0	0.5	0.0240(3)	1.0000	0.84(8)
Li2 (T5)	8*g*	0.8168(4)	0.23816(4)	0.2232(3)	1.0000	0.94(5)
Li3 (T3)	2*d*	0	0.5	0.75	1.0000	1.52(5)

### 3.3 Room- and Low-Temperature Structure Descriptions

#### 3.3.1 Li_7_TiO_5_X (X = Cl^–^, Br^–^)—Room-Temperature Structures

Li_7_TiO_5_Br and Li_7_TiO_5_Cl adopt the cubic argyrodite structure (*F*4̅3*m*) at room temperature (Figure 4a,b) comparable to the crystal structures of other oxide and sulfide argyrodites (Figure S1).^15^ The X^–^ and O^2–^ anions form a tetrahedrally close-packed structure in which the Ti^4+^ and Li^+^ cations occupy a proportion of the tetrahedral holes, e.g., Ti^4+^ ions (4*a*) are coordinated by four O^2–^ ions (16*e*), defining isolated TiO_4_^4–^ tetrahedra (Figure 4a–c).

**Figure 4:**
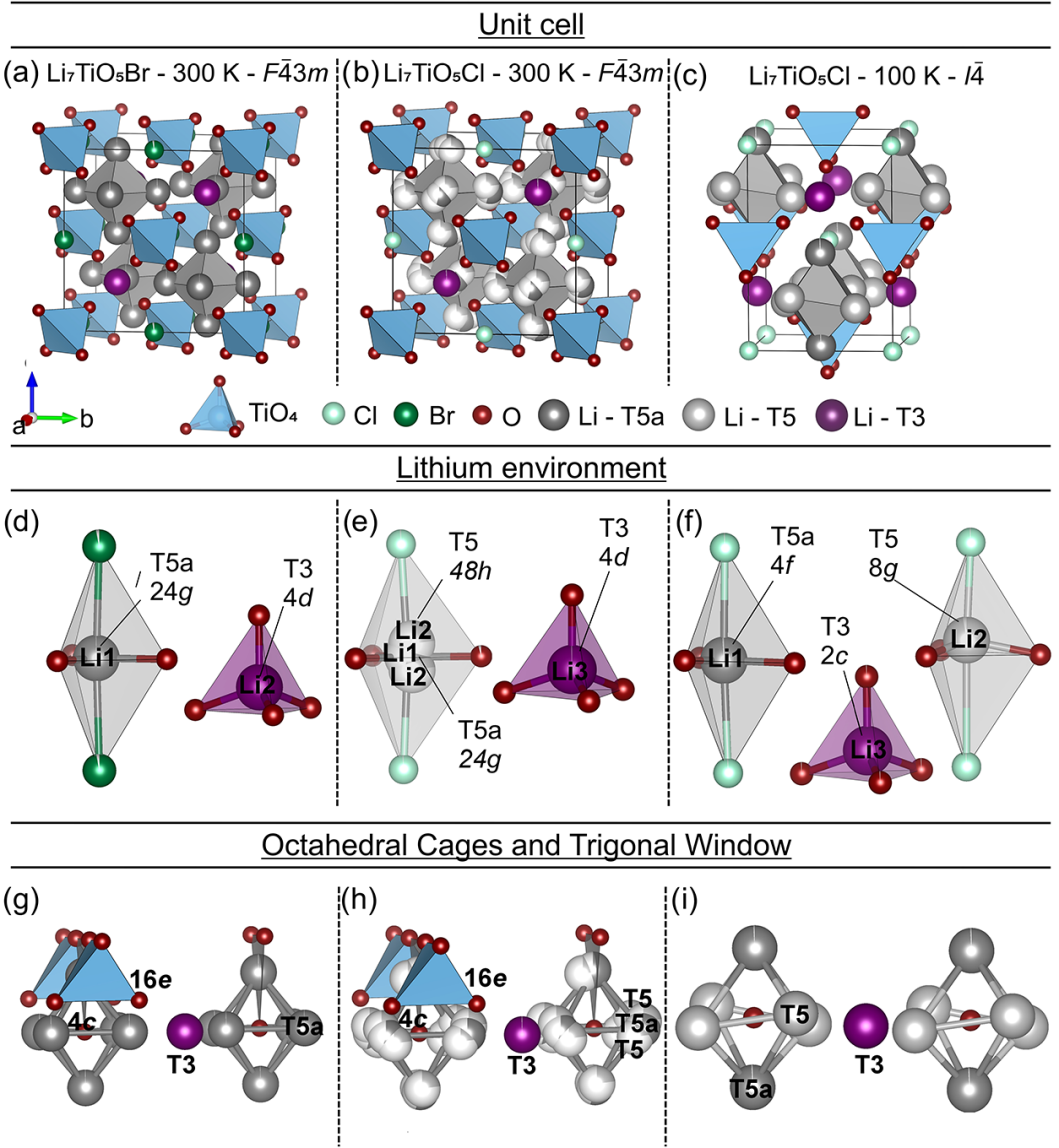
(a–c) Unit cells for (a) Li_7_TiO_5_Br-300 K, (b) Li_7_TiO_5_Cl-300 K, and (c) Li_7_TiO_5_Cl-100 K. (d–f) Lithium environments highlighting the different (T5, T5a, and T3) sites which are either fully or partially occupied giving rise to order/disorder and symmetry changes in (d) Li_7_TiO_5_Br-300 K, (e) Li_7_TiO_5_Cl-300 K, and (f) Li_7_TiO_5_Cl-100 K. (g–i) Octahedral cages highlighting occupied Li^+^ sites as well as triangular bottle necks in (g) Li_7_TiO_5_Br-300 K, (h) Li_7_TiO_5_Cl-300 K, and (i) Li_7_TiO_5_Cl-100 K.

In Li_7_TiO_5_Br, Li^+^ ions occupy the trigonal bipyramidal T5a (24*g*) and tetrahedral T3 (4*d*) sites to give an ordered Li^+^ site distribution (Figure 4d), similar to that of Li_6_PO_5_Br.^25^ Occupancy of the T5a sites produces octahedral cages of Li^+^ ions that surround central oxide anions (4*d*) (Figure 4g).

In Li_7_TiO_5_Cl, Li^+^ ions occupy the trigonal bipyramidal T5a (24*g*), and tetrahedral T5­(48*h*) and T3 (4*d*) sites (Figure 4e), which is one additional site beyond Li_7_TiO_5_Br. The T5 and T5a sites are partially occupied with *s.o.f.* of 0.6013(14) and 0.199(7), respectively, while the T3 site is fully occupied, giving a disordered Li^+^ site distribution (Figure 4h). Such Li^+^ site disorder on the T5a and T5 sites within the octahedral cages has been linked to high ionic conductivities in oxide and sulfide argyrodites such as Li_6+*x*_P_1–*x*_Si_*x*_O_5_Cl^26^ and Li_6+*x*_Si_*x*_Sb_1–*x*_S_5_I.^11^

#### 3.3.2 Li_7_TiO_5_Cl—Low-Temperature Structure

Below 250 K, the Li_7_TiO_5_Cl structure transitions from cubic *F*4̅3*m* to tetragonal *I*4̅ symmetry (Figure 3, 4). The unit cells of Li_7_TiO_5_Cl at 300 and 100 K are compared in Figure 5, with the cubic unit cell depicted as dashed lines in Figure 5d–f to facilitate the comparison between the two symmetries. The poly­(anion) structure remains largely unchanged through the transition, and the reduction in symmetry originates from changes to the Li^+^ sites, similar to order–disorder transitions known for other argyrodites.^28,44^ A subset of the T5a and T5 sites that are partially occupied at 300 K in Li_7_TiO_5_Cl (Figure 4) becomes fully occupied at 100 K; 25% of the available T5 (8*g*) sites and 50% of the available T5a (4*f*) sites are fully occupied, while the T3 (2*c*) position remains fully occupied at both temperatures (Figure 4f). This results in fully ordered Li^+^ sites at 100 K, distorting the Li^+^ octahedral cages (Figures 4i and 5d–f) and breaking symmetry elements such as the 3-fold rotational axis along the body diagonal (Figure 5b,e) and mirror plane along the face-diagonal (Figure 5c,f) of the unit cell, reducing the symmetry to tetragonal *I*4̅.

Order–disorder transitions of this kind are well-documented for many argyrodite materials, even those beyond Li^+^. Numerous ordered cubic, orthorhombic, and monoclinic structures have been reported for argyrodites, such as Li_7_SiO_5_Cl (*P*2_1_3),^26^ Li_7_PS_6_ (*Pna*2_1_),^28^ and Li_6_PS_5_I (*Cc*).^44^ However, tetragonal symmetry has been observed only for one other argyrodite: Li_7_Zn_0.5_SiS_6_.^45^ This sulfide argyrodite crystallizes in the same *I*4̅ space group at room temperature, albeit with a significantly larger unit cell, which results from a complex ordering pattern and undergoes a phase transition at 411 K to *F*4̅3*m* symmetry. This highlights the potential structural diversity in lithium argyrodites that remains unexplored, which is further emphasized by noting that the ordering behavior observed for Li_7_TiO_5_Cl accesses a structure that is distinct from the ordered cubic structure of Li_7_TiO_5_Br, which likely results from lattice pressure induced by the different ionic radii of the halide anions. Further exploration of these systems may include the substitution of F^–^ at the Cl^–^/Br^–^ or even O^2–^ sites, with similar ionic radius (*r*_O_: 1.38 Å vs r_F_: 1.31 Å),^45^ to access compositions with reduced Li content and to induce anion site disorder that may enable enhanced control over the ionic conductivity of these materials.

### 3.4 Transport Properties

The transport properties of Li_7_TiO_5_X (X = Cl^–^, Br^–^) were investigated using AC impedance spectroscopy at and above room temperature, coupled with DC polarization measurements and NMR spectroscopy. Pellets utilized in AC Impedance measurements were prepared via reactive sintering, resulting in densities of 80% and 81% of the theoretical densities for Li_7_TiO_5_Cl and Li_7_TiO_5_Br, respectively. A typical Nyquist plot collected for Li_7_TiO_5_Cl at room temperature is shown in Figure 6a. The plot contains a single semicircle and a low-frequency spike, a characteristic feature of an ion conductor arising from the buildup of charge at the ion-blocking electrode caused by the movement of Li^+^ ions. The capacitance at the frequency of maximum loss was calculated to be 2.11 pF, indicating that the signal corresponded to a bulk response (Figure S4). The Nyquist data were therefore modeled using a resistor in parallel with a constant phase element (CPE), integrated in series with another CPE to account for the low-frequency spike. The ionic conductivity of Li_7_TiO_5_Cl, calculated from the low-frequency intercept of the semicircle assigned to the bulk contribution of the material, was measured to be 2.2(2) × 10^–6^ S cm^–1^ at room temperature. Nyquist plots collected for Li_7_TiO_5_Br (Figure S3a) reveal a very low room-temperature ionic conductivity of ∼10^–9^ S cm^–1^, 3 orders of magnitude lower than that of Li_7_TiO_5_Cl.

Both materials follow Arrhenius-type behavior (Figure 6b) at elevated temperatures, characterized by activation energies of 0.36(2) eV and 0.58(2) eV for Li_7_TiO_5_Cl and Li_7_TiO_5_Br, respectively. A change in slope is clearly observed in the Arrhenius plot of Li_7_TiO_5_Cl below room temperature (Figure S5), which can be attributed to the Li^+^ site ordering observed in the *I*4̅ structure. An increased activation energy of 0.509(3) eV is obtained in the temperature range of 276–233 K, which is more closely comparable with the activation energy of 0.58(2) eV extracted above room temperature from the Li^+^ site-ordered *F*4̅3*m* structure of Li_7_TiO_5_Br, highlighting the impact of Li^+^ site order vs disorder within these materials. XRD patterns collected from a ground pellet after the AC impedance measurements show no changes, confirming the stability of the Li_7_TiO_5_Cl under the measurement conditions (Figure S6). Measurement of DC polarization data at room temperature indicates that the electronic contribution to the total conductivity is negligible at 1%. for Li_7_TiO_5_Cl. (Figure S3c,d). This low electronic contribution is further supported by the measurement of a wide band gap of 4.29(7) eV for Li_7_TiO_5_Cl (Figure S7).

Static ^7^Li solid-state NMR spectra for Li_7_TiO_5_Cl (Figure 6c) and Li_7_TiO_5_Br (Figure S3b) in the 190–588 K temperature range enable detection of Li-ion dynamics on the kHz time scale and are complementary to AC impedance measurement. The central transition detected in the spectrum acquired for Li_7_TiO_5_Cl at 190 K is characterized by a full width at half-maximum in the rigid lattice regime, Δν_R_, of ∼9.9 kHz and exhibits a predominantly Gaussian line shape dominated by ^7^Li–^7^Li homonuclear dipole interactions (Figure 6c). As the temperature is increased above ∼247 K (onset temperature *T*_onset_ in Figure 6d), line narrowing of the ^7^Li central transition for Li_7_TiO_5_Cl is observed, which reveals an increase in Li^+^ motion at this temperature. Above ∼425 K, a predominantly Lorentzian line shape and line width in the fast motional regime Δν_∞_ lower than 570 Hz are observed, indicating that the ^7^Li–^7^Li homonuclear dipole interactions are fully averaged by Li^+^ ion motion. Fitting a Boltzmann sigmoid function to the Δν­(*T*) vs *T* line narrowing curve^46^ (Figure 6d) enables extraction of a mean Li-ion jump rate of τ^–1^ = 2πΔν_R_ = 6.24 × 10^4^ s^–1^ for Li_7_TiO_5_Cl at the observed inflection point of 247 K.

**Figure 5:**
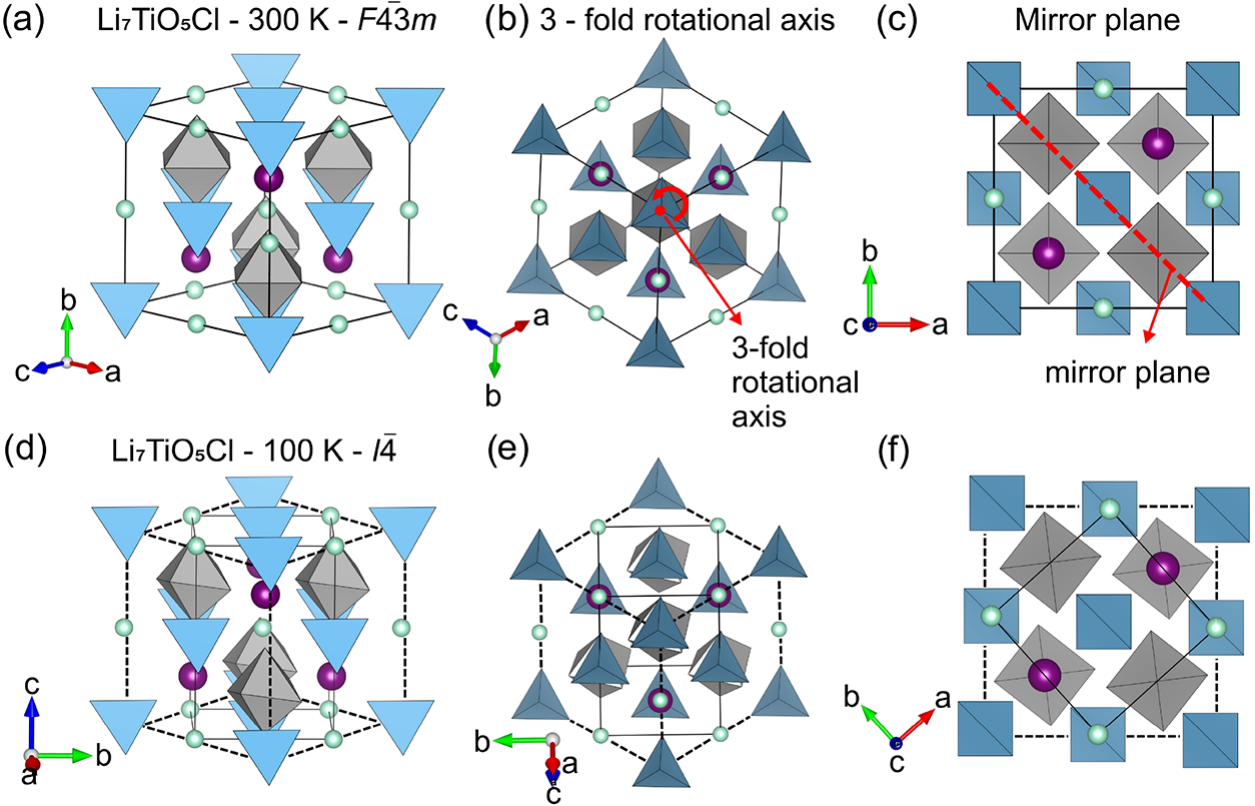
Comparison between the unit cell of Li_7_TiO_5_Cl at (a) 300 K and (d) 100 K. Ordering of Li^+^ ions onto 25% of the available T5 sites leads to distorted Li^+^ octahedra, which breaks the 3-fold rotational axis (b, e) and mirror plane (c, f) symmetry operators of *F*4̅3*m* symmetry. The higher-symmetry cubic unit cell is drawn with dashed lines in (d–f) to aid comparison, and symmetry elements are drawn in red. Atom colors: TiO_4_ tetrahedra (light blue), OLi_6_ octahedra (light gray), Cl^–^ (turquoise), Li^+^ (T3) (purple)*:*.

The ^7^Li central transition of Li_7_TiO_5_Br at 205 K has a predominantly Gaussian line shape with Δν_R_ of ∼10.5 kHz at this temperature (Figure S3b), which narrows significantly as the temperature is increased, revealing a two-component line shape in spectra acquired in the 329–449 K temperature range (Figure S3b), attributed to two distinct Li^+^ environments that each undergo motional processes in different temperature ranges. The narrow component observed in Li_7_TiO_5_Br has a constant Δν within this temperature range and is tentatively assigned to the small percentage of Li^+^ cations in the fast motional regime occupying the T3 position that are likely involved in localized Li-ion motional processes. The dominant broader component is observed to undergo line narrowing at higher temperatures and is assigned to the larger percentage of Li^+^ cations in the T5a position, with a Lorentzian line shape and line width Δν_∞_ lower than 725 Hz observed above 569 K. Despite the multicomponent contribution in Li_7_TiO_5_Br, comparison of the observed temperature dependence is indicative of overall enhanced Li-ion dynamics in Li_7_TiO_5_Cl, in accordance with the measured impedance data.

**Figure 6:**
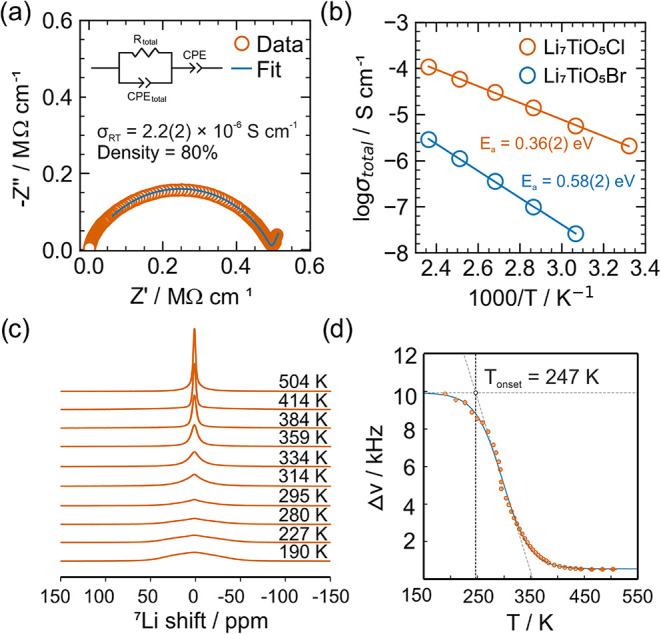
(a) Typical Nyquist plot for Li_7_TiO_5_Cl at room temperature, with the equivalent circuit used to fit the data inset. (b) Arrhenius plot for Li_7_TiO_5_Cl (orange) and Li_7_TiO_5_Br (blue) with extracted activation energies. (c) ^7^Li variable-temperature NMR spectra of Li_7_TiO_5_Cl recorded at 9.4 T under static conditions. (d) Temperature-dependent full width at half-maximum of the static ^7^Li NMR central transition, Δν­(*T*), measured for Li_7_TiO_5_Cl at 9.4 T, highlighting the onset temperature, *T*_onset_, of line narrowing (vertical, dashed line).

### 3.5 Structure–Property Relationship

Three dominant local Li^+^ ion jumps have been identified in lithium argyrodites: doublet jumps between T5 sites within one trigonal bipyramidal unit via a trigonal window defined by three anions, intracage jumps within one octahedral cage, and intercage jumps connecting neighboring octahedral cages, all of which are important for long-range lithium mobility (Figure 7a). Enhanced lithium diffusion can be facilitated by the shortening of these inter- and intracage distances, increased Li^+^ site disorder, enhanced lithium content, and improved mobility between neighboring sites via the optimization of anionic windows that the Li^+^ ions traverse.^14,47–49^

**Figure 7:**
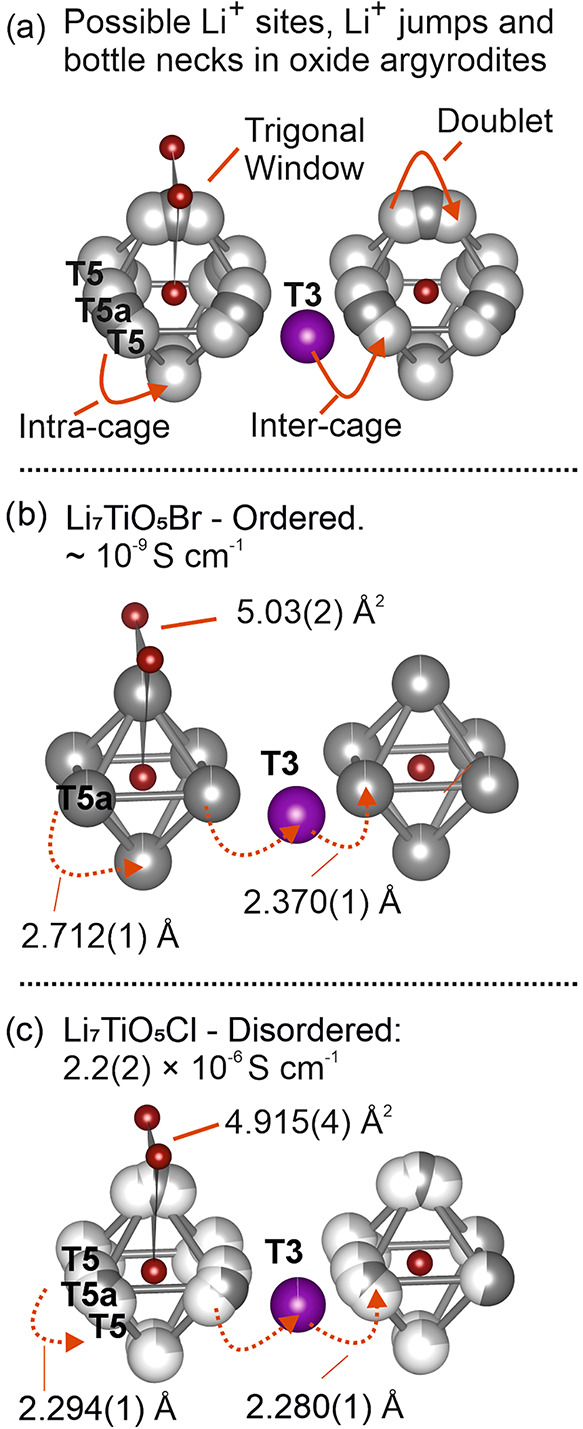
(a) Octahedral Li^+^ cages in argyrodites highlighting possible Li^+^ ion sites, intra- and intercage jumps, and the trigonal window through which Li^+^ ions traverse. (b) Octahedral cage in the Li-site-ordered Li_7_TiO_5_Br and its intra- and intercage distances and the anion window size. (c) Jump distances are significantly smaller in the Li-site disordered Li_7_TiO_5_Cl resulting in a 1000-fold increase in ionic conductivity compared to Li_7_TiO_5_Br.

Li_7_TiO_5_Br has a low ionic conductivity (∼10^–9^ S cm^–1^) and high activation energy (0.58(2) eV), comparable to the values reported for Li_6_PO_5_Br (∼10^–9^ S cm^–1^, ∼0.60 eV)^25^ (Table 2). This results from large intra- and intercage jump distances of 2.712(1) Å (T5a-T5a) and 2.370(1) Å (T5a-T3), respectively (Figure 7b, and Table 2). These are driven by the unit cell expansion due to the larger ionic radius of Br ions as well as the ordered Li^+^ site distribution with fully occupied T5a and T3. The occupancy of Li­(T5a) sites in Li_7_TiO_5_Br is similar to that of Li_6_PO_5_Br; however, occupancy of the additional T3 site in Li_7_TiO_5_Br offers a shorter Li–Li distance of 2.370(1) Å that connects separate cages via T5a–T3–T5a jumps (Figure 7b) compared to the intercage distance of 3.155(4) Å in Li_6_PO_5_Br (Table 2). The trigonal oxide anion window defining the doublet jump is comparable in Li_6_PO_5_Br (5.014 Å^2^) to Li_7_TiO_5_Br (5.03(2) Å^2^).

**Table 2:** Intra- and Intercage Distances, Trigonal Window Sizes, Activation Energies (*E*_a_) and Room-Temperature Ionic Conductivities (σ_RT_) in Selected Oxide Argyrodites

compound	intracage jump/Å	intracage distance/Å	intercage jump/Å	intercage distance/Å	trigonal window size/Å^2^	E_a_ / eV	σ_RT_ / S cm^–1^
Li_6_PO_5_Br^25^	T5a–T5a	2.737(9)	T5a–T5a	3.155(4)	5.014(3)	∼ 0.60	∼ 10^–9^
Li_6_PO_5_Cl^25^	T5a–T5a	2.728(9)	T5a–T5a	3.087(9)	4.856(5)	∼ 0.60	∼ 10^–9^
Li_7_TiO_5_Br [this work]	T5a–T5a	2.712(1)	T5a–T3–T5a	2.370(1)	5.03(2)	0.58(2)	∼ 10^–9^
Li_6.75_P_0.25_Si_0.75_O_5_Cl^26^	T5–T5	2.101(13)	T5–T4–T5	1.606(3)	4.827(4)	0.52(2)	1.82(1) × 10^–6^
Li_7_TiO_5_Cl [this work]	T5–T5	2.294(1)	T5a–T3–T5a	2.280(1)	4.915(4)	0.36(2)	2.2(2) × 10^–6^

Li_7_TiO_5_Cl exhibits an ionic conductivity of 2.2(2) × 10^–6^ S cm^–1^, three orders of magnitude higher than Li_7_TiO_5_Br, which results from its enhanced Li^+^ site disorder. This is also reflected in the 0.36(2) eV activation energy of Li_7_TiO_5_Cl, which is significantly lower than 0.58(2) eV for Li_7_TiO_5_Br and 0.52(2) eV for Li_6.75_P_0.25_Si_0.75_O_5_Cl.^26^ In fact the measured activation energy of 0.36(2) eV for Li_7_TiO_5_Cl is comparable to those measured for some sulfide Li_6_PS_5_X (X = Cl^–^, Br^–^) materials.^50,51^ Li_7_TiO_5_Cl exhibits a high room-temperature ionic conductivity (2.2(2) × 10^–6^ S cm^–1^) and the lowest activation energy for bulk Li^+^ ion motion (0.36(2) eV) reported for all oxide argyrodites.

This performance is a direct consequence of the Li^+^ site disorder present in Li_7_TiO_5_Cl, and high lithium content (7 Li^+^ per formula unit). Occupancy of the T5a, T5, and T3 sites by Li^+^ in Li_7_TiO_5_Cl enables access to much shorter intra- and intercage distances compared to Li_7_TiO_5_Br, facilitating fast Li^+^ ion diffusion (Figure 7c and Table 2) and underscoring the pivotal role of site disorder within oxide argyrodites. Through consideration of these Li–Li distances alone, it is clear that the disordered *F*4̅3*m* structure of Li_7_TiO_5_Cl facilitates extended Li^+^ ion diffusion through an interconnected network of Li^+^ sites in three dimensions (Figure S8). Bond valence site energy (BVSE) calculations confirm this, revealing a 3D percolation network with inter- and intracage jumps as often reported for Li-sulfide argyrodites (Figure S9). Specifically, the BVSE calculations indicate that the intracage jumps (T5–T5) are rate-limiting compared to lower-energy more favorable intercage jumps (T5–T3). Li_6.75_P_0.25_Si_0.75_O_5_Cl, which exhibits a comparable room-temperature ionic conductivity (1.82(1) × 10^–6^ S cm^–1^) to Li_7_TiO_5_Cl, also has extensive Li^+^ site disorder with occupancy of the T5, T5a, T3, and T4 sites. Occupancy of the T4 site in Li_6.75_P_0.25_Si_0.75_O_5_Cl, which is unoccupied in Li_7_TiO_5_Cl, enables access to alternative intercage pathways (T5–T4–T5) with a shorter distance of 1.606(3) Å compared to the shortest intercage (T5a–T3–T5a) pathway in Li_7_TiO_5_Cl with distances of 2.280(1) Å (Table 2). Despite this, Li_7_TiO_5_Cl exhibits a slightly higher ionic conductivity and much lower activation energy, which likely results from the combination of increased Li^+^ carrier concentration (7 Li^+^ per formula unit) and the larger trigonal windows through which Li^+^ ions traverse. The trigonal window defined by oxide anions in Li_7_TiO_5_Cl (4.915(4) Å^2^) is larger than that of Li_6.75_P_0.25_Si_0.75_O_5_Cl (4.827(4) Å^2^) as a result of the increased unit cell size expanded by the incorporation of larger Ti^4+^, and likely facilitates higher Li^+^ ion mobility. This demonstrates that changes to the local Li^+^ motion within the T5/T5a octahedral cages via control of anionic window sizes alongside an increase lithium content, maximized to 7 Li^+^ per formula unit in this case, can influence long-range Li^+^ ion transport in these particular oxide argyrodites.

## 4 Conclusions

This study presents the synthesis, crystal structures, and transport properties of Li_7_TiO_5_X (X = Cl^–^, Br^–^), the first lithium argyrodites in which the framework-forming cation is a transition metal, highlighting the expansive chemical space that is accessible for oxide argyrodite chemistry. Incorporation of a large tetrahedron-forming cation with + IV oxidation state (Ti^4+^) allows us to maximize the lithium content to 7 Li^+^ per formula unit in the absence of Li^+^ site ordering in Li_7_TiO_5_Cl, which is common for high-lithium-content argyrodites. Li_7_TiO_5_Cl adopts a Li^+^ site disordered cubic *F*4̅3*m* structure at room temperature with partial occupancy of T5a and T5 sites alongside full occupancy of T3 sites. This disorder facilitates high Li^+^ ion mobility with a measured ionic conductivity of 2.2(2) × 10^–6^ S cm^–1^ at room temperature and the lowest reported activation energy of 0.36(2) eV for bulk lithium transport in any oxide argyrodite material. In contrast, Li_7_TiO_5_Br adopts a Li^+^ site-ordered cubic *F*4̅3*m* structure with fully occupied T5a and T3 sites and has a much lower ionic conductivity (∼10^–9^ S cm^–1^) and significantly higher activation energy (0.58(2) eV). Interestingly, Li_7_TiO_5_Cl exhibits an order–disorder phase transition below room temperature; however, adopting a more complex Li^+^ site ordering pattern than Li_7_TiO_5_Br that yields a tetragonal superstructure with *I*4̅ symmetry below 250 K. The low-temperature structure of Li_7_TiO_5_Cl represents only the second time that a tetragonal argyrodite structure has been observed, further emphasizing the potential for greater structural diversity in argyrodite materials that may be accessed by expanding the range of possible framework-forming cation chemistry.

## Supplementary Material



## Data Availability

The data that support the findings of this research will be made openly available via the University of Liverpool open research platform Liverpool Elements on acceptance of the publication.

## Notes

ICP-OES and IC results for Li_7_TiO_5_Cl and Li_7_TiO_5_Br, additional Rietveld refinement models tested during structure determination, details of normal mode calculations, and impedance and ^7^Li NMR data of Li_7_TiO_5_Br (PDF)
