# Supplementary material for: High Lithium Content and Site Disorder in the Transition Metal Oxide Argyrodites Li7TiO5X (X = Cl–, Br–)
Source: Chem Mater. 2026 Jun 22;38(13):6312–22. doi: 10.1021/acs.chemmater.6c00177 (PMC13374010; doi:10.1021/acs.chemmater.6c00177)
Supplement: Supplementary file 1 [file cm6c00177_si_001.pdf]

Supplementary Information

High Lithium Content and Site Disorder in the Transition Metal Oxide Argyrodites Li_7TiO_5X (X = Cl^-, Br^-)

Alexandra Morscher,[a] Lucia Corti,[a,b] Samuel L. Goodwin,[a] Andrés Acín-Lalanza,[a] Matthew A. Wright,[a,c] T. Wesley Surta,[a] Ruiyong Chen,[a] Matthew S. Dyer, [a,b] Frédéric Blanc,[a,b,c] Luke M. Daniels,[a] John B. Claridge[a,b] and Matthew J. Rosseinsky*[a,b]

[a] Department of Chemistry, University of Liverpool, Crown Street, L69 7ZD Liverpool, UK.

[b] Leverhulme Research Centre for Functional Materials Design, Materials Innovation Factory, 51 Oxford Street, University of Liverpool, Liverpool L7 3NY, UK.

[c] Stephenson Institute for Renewable Energy, University of Liverpool, Peach Street L69 7ZF Liverpool, UK.

*Corresponding Author: m.j.rosseinsky@liverpool.ac.uk

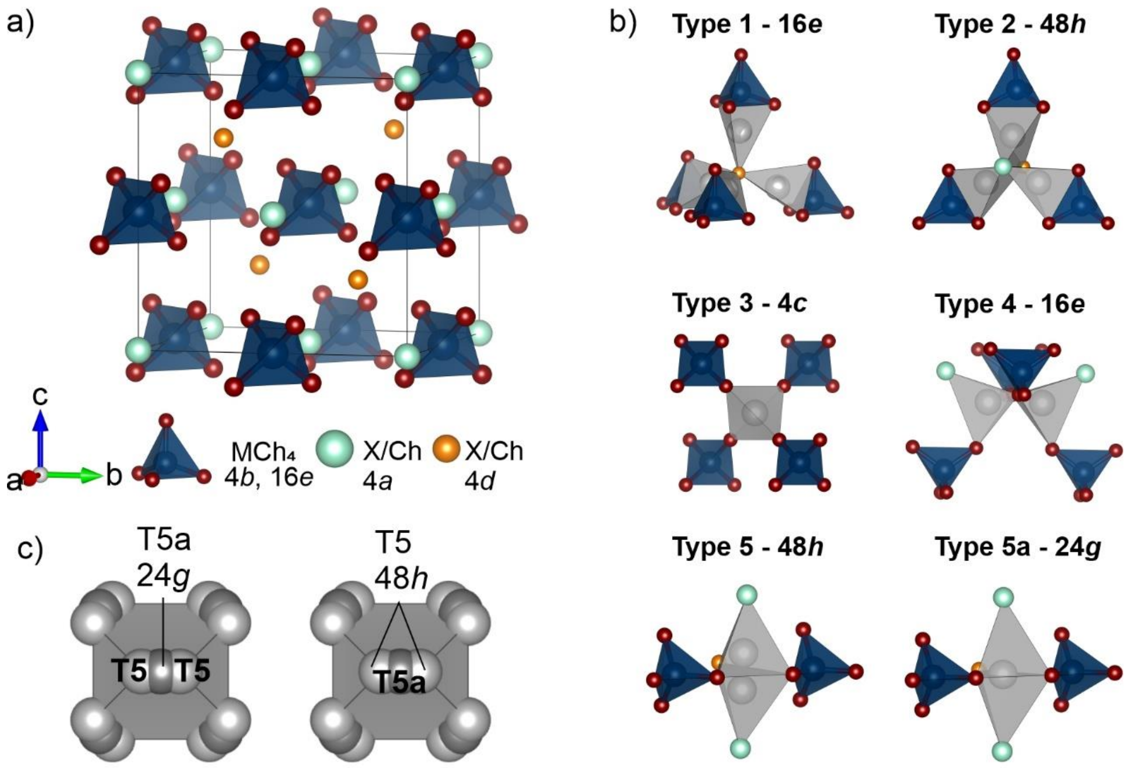

Figure S1: (a) Unit cell of $Li_{7-x+y}M^{5-y}Ch_{7-x}X_x$ (*M* = P^{5+}, Sb^{5+}, Si^{4+} and Ge^{4+}; *Ch* = O^{2-}, S^{2-}, and Se^{2-}; *X* = Cl^-, Br^-, and I^-); *Ch/X* anions are tetrahedrally close packed on Wyckoff site (4a, 4d, and 16e) forming 136 tetrahedral voids; four are occupied by *M* cations on the 4b site defining *MCh₄* tetrahedra (Ch: 16e). Lithium atoms are not shown. (b) Panels showing five types (T1, T2, T3, T4, and T5) of possible interstitial tetrahedral sites available for lithium occupancy and the trigonal bipyramidal T5a site (classification proposed in ref (1)). Lithium atoms are shown in grey. (c) Octahedral Li^+-ion cages consisting of T5 and T5a sites surrounding central anions on the 4d site; due to the close proximity of T5 and T5a sites, the total occupancy over the two sites is constrained to unity. Reproduced with permission from Chem.–Eur. J.**2010,**16 (7), 2198–2206 (ref (1).). Copyright 2010 John Wiley and Sons.[1]

Table S1: Empirical formula determined for Li_7TiO_5Cl and Li_7TiO_5Br from ICP-OES (inductively coupled plasma-optical emission spectroscopy) and IC (ion chromatography) compositional analysis. Measurements were made in triplicate and corrected using appropriate standards.

Nominal Formula	Li	Ti	Halide (Cl/Br)
	Li_7TiO_5Cl		
Mass content (mg L^{-1})	18.524	21.407	16.113
Standard deviation (mg L^{-1})	0.162	0.049	0.150
Correction factor	1.1327	0.9918	0.9852
Normalised element content	6.855	1.000	0.968
Error	0.066	0.034	0.009
	Li_7TiO_5Br		
Mass content (mg L^{-1})	18.270	20.786	41.147
Standard deviation (mg L^{-1})	0.166	0.082	0.461
Correction factor	1.1327	0.9918	0.9717
Normalised element content	6.963	1.000	1.087
Error	0.045	0.054	0.012

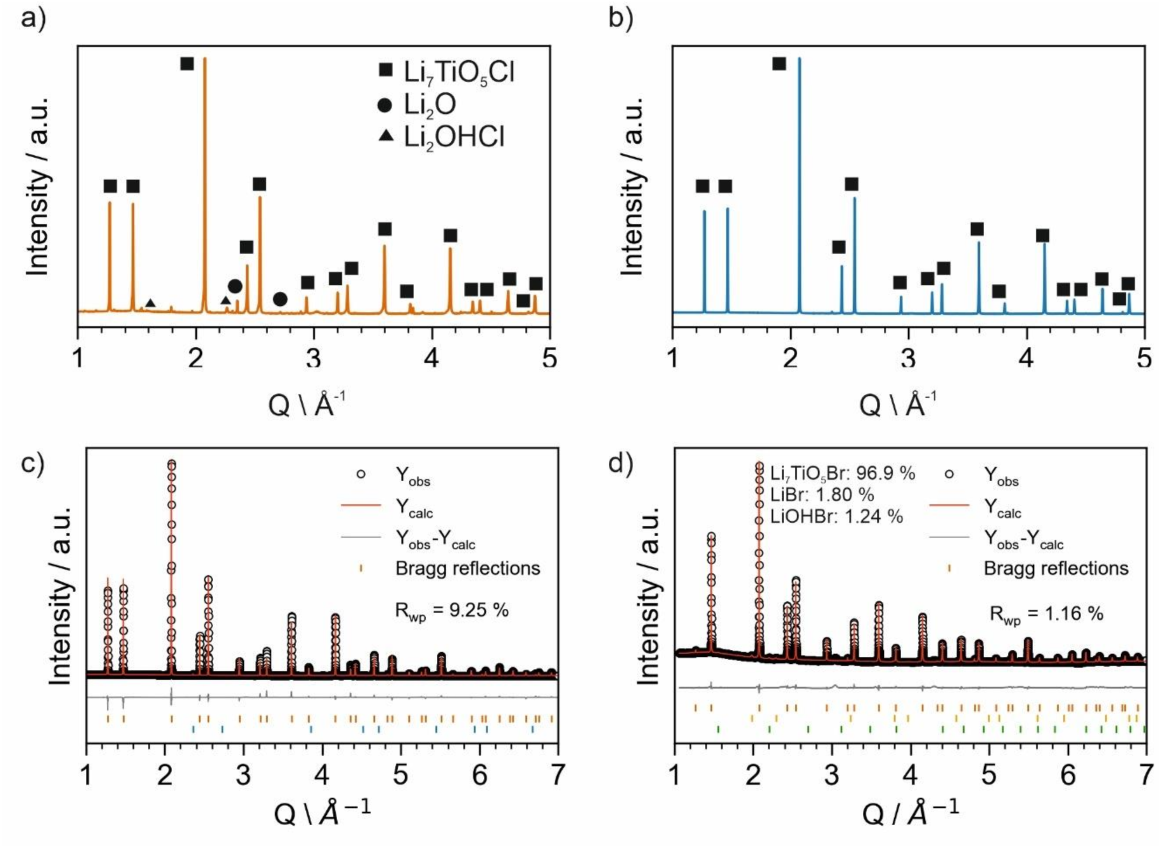

Figure S2: a) PXRD data collected from a sample of Li_7TiO_5Cl synthesised at 823 K which contains impurities of Li_2O, Li_2OHCl and other unknown impurities, compared against a phase pure sample of b) Li_7TiO_5Cl synthesised at 898 K. c) Rietveld refinement of a structural model of Li_7TiO_5Cl against high resolution synchrotron XRD data at 300 K without Li-site disorder (blue tick marks are for Li_2O). d) Rietveld fit of Li_7TiO_5Br against high resolution synchrotron XRD data at 100 K, Y_{obs} (black circles), Y_{calc} (red line), Y_{obs}–Y_{calc} (grey line) and Bragg reflections (orange tick marks for Li_7TiO_5Br 96.9 wt%, yellow tick marks for LiBr 1.80 wt%, green tick marks for Li_2OHBr 1.24 wt%).

Table S2. The normal mode frequencies, v, calculated using density functional theory (DFT) for the $F\bar{4}3m$ structure of Li_7TiO_5Cl. The 15 modes from 154 to 168 have imaginary frequencies and represent unstable modes. These fall in four symmetrically unique groups, 154–156, 157–162, 163–165 and 166–168. One representative mode was selected from each of these groups to generate a structure in reduced symmetry to perform further geometry optimisation calculations.

Mode	v/cm^{-1}	Mode	v/cm^{-1}	Mode	v/cm^{-1}	Mode	v/cm^{-1}	Mode	v/cm^{-1}	Mode	v/cm^{-1}
1	828.8	29	628.1	57	472.6	85	377.7	113	254.5	141	139.4
2	828.8	30	628.1	58	472.6	86	377.7	114	254.5	142	92.8
3	828.8	31	628.1	59	472.6	87	377.7	115	254.5	143	92.8
4	823.0	32	628.1	60	472.6	88	377.7	116	254.5	144	92.8
5	823.0	33	540.2	61	472.6	89	369.1	117	254.5	145	62.4
6	823.0	34	540.2	62	462.7	90	369.1	118	235.9	146	62.4
7	800.3	35	540.2	63	462.7	91	369.1	119	235.9	147	62.4
8	735.7	36	505.9	64	462.7	92	351.2	120	235.9	148	62.4
9	735.7	37	505.9	65	435.7	93	351.2	121	213.8	149	62.4
10	735.7	38	505.9	66	435.7	94	345.5	122	213.8	150	62.4
11	735.3	39	501.4	67	435.7	95	345.5	123	213.8	151	0.6
12	735.3	40	501.4	68	433.2	96	345.5	124	208.2	152	0.6
13	735.3	41	501.4	69	433.2	97	312.6	125	208.2	153	0.6
14	735.3	42	501.4	70	433.2	98	312.6	126	208.2	154	60.6i
15	735.3	43	501.4	71	426.1	99	312.6	127	188.9	155	60.6i
16	735.3	44	501.4	72	426.1	100	297.4	128	188.9	156	60.6i
17	638.0	45	496.5	73	426.1	101	297.4	129	188.9	157	100.1i
18	638.0	46	496.5	74	426.0	102	297.4	130	163.8	158	100.1i
19	638.0	47	496.0	75	426.0	103	297.4	131	163.8	159	100.1i
20	631.4	48	496.0	76	426.0	104	297.4	132	163.8	160	100.1i
21	631.4	49	496.0	77	426.0	105	297.4	133	163.8	161	100.1i
22	631.4	50	491.1	78	426.0	106	290.2	134	163.8	162	100.1i
23	629.2	51	491.1	79	426.0	107	290.2	135	163.8	163	121.2i
24	628.4	52	491.1	80	396.2	108	290.2	136	139.4	164	121.2i
25	628.4	53	478.3	81	396.2	109	274.1	137	139.4	165	121.2i
26	628.4	54	478.3	82	396.2	110	274.1	138	139.4	166	133.7i
27	628.1	55	478.3	83	377.7	111	274.1	139	139.4	167	133.7i
28	628.1	56	472.6	84	377.7	112	254.5	140	139.4	168	133.7i

Table S3. The space groups and relative energies of the four DFT geometry optimised structures of Li_7TiO_5Cl generated from representative unstable normal modes with imaginary frequencies given in Table S2. The space group of the optimised structures was determined using Findsym.[2]

Unstable Mode	Space group	Energy (eV/formula unit)
	$F\bar{4}3m$	-81.1847
156	$P1$	-81.2774
162	$P1$	-81.2774
165	$P\bar{4}n2$	-81.2406
168	$I\bar{4}$	-81.2775

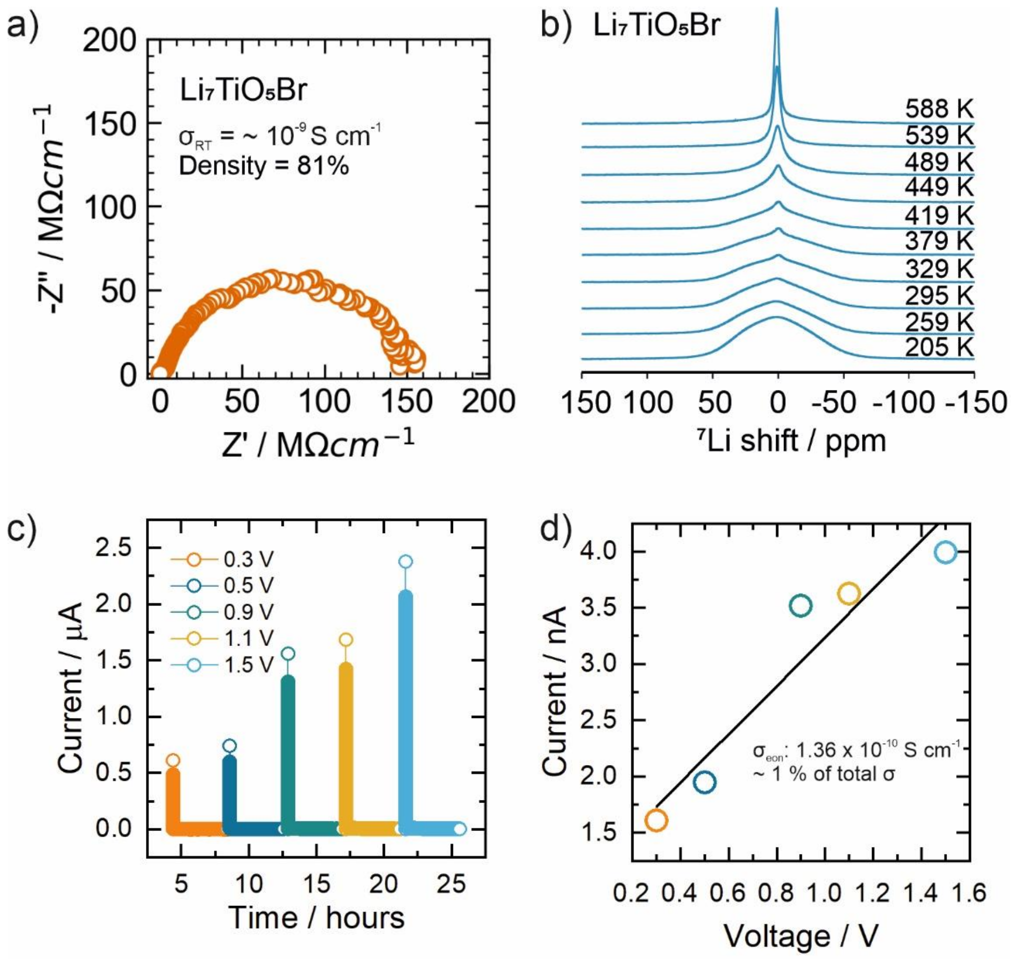

Figure S3: a) Nyquist plot for Li_7TiO_5Br at room temperature, b) ^{7}Li variable-temperature NMR spectra of Li_7TiO_5Br recorded at 9.4 T under static conditions, c-d) DC polarisation for Li_7TiO_5Cl confirming a low electronic contribution (< 1 %) to the total ionic conductivity measured via AC Impedance spectroscopy

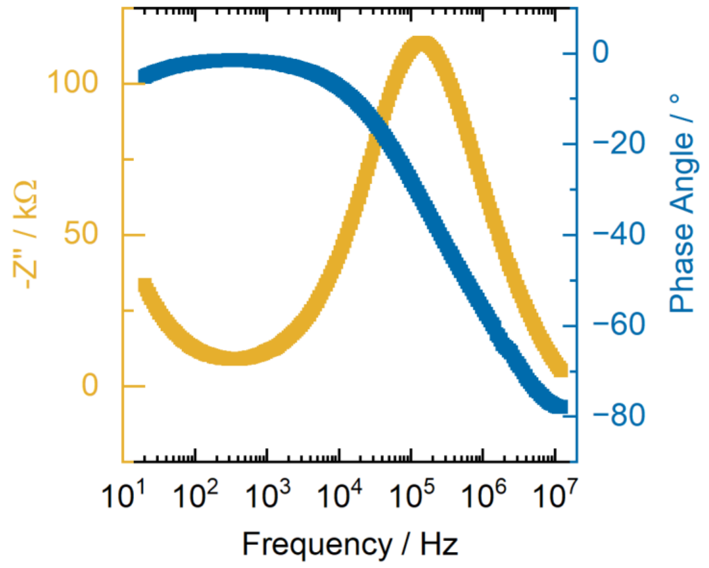

Figure S4: Bode Plot for Li_7TiO_5Cl showing a single contribution to the impedance spectra, which was assigned to the bulk response using its capacitance value of 2.11 pF

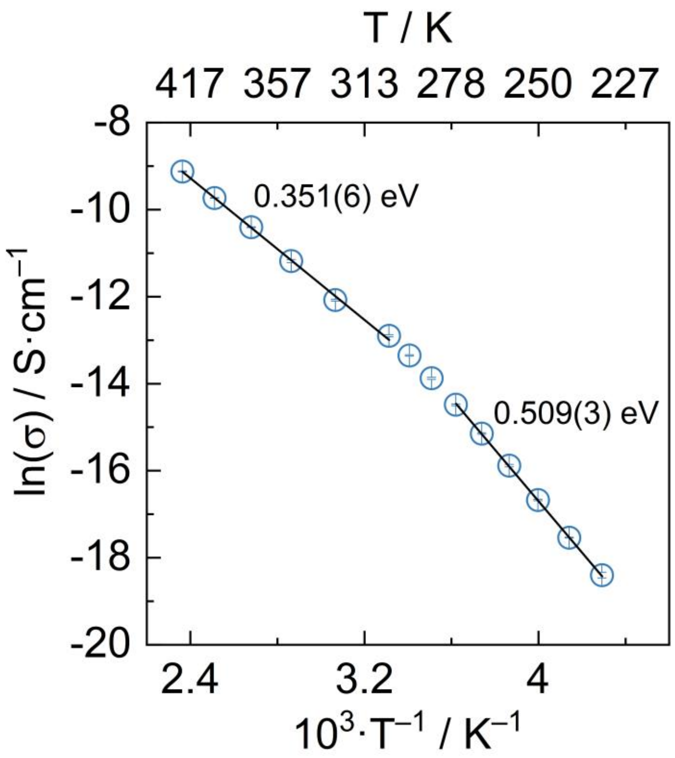

Figure S5: Temperature dependence of the total conductivity in the range 423-233 K in the form of an Arrhenius plot for Li_7TiO_5Cl with extracted activation energies shown.

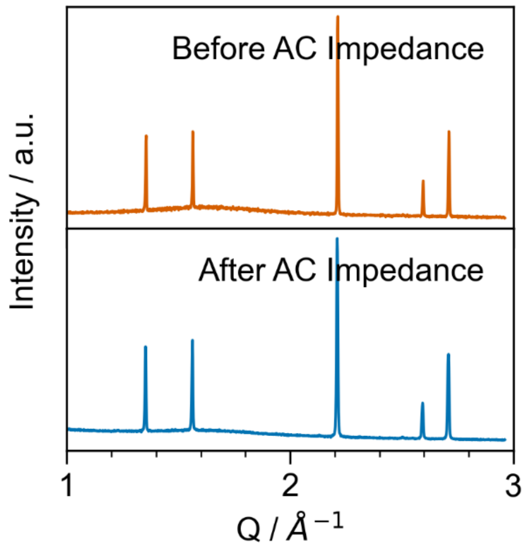

Figure S6: XRD patterns of Li_7TiO_5Cl collected before (upper) and after (lower) AC impedance measurement.

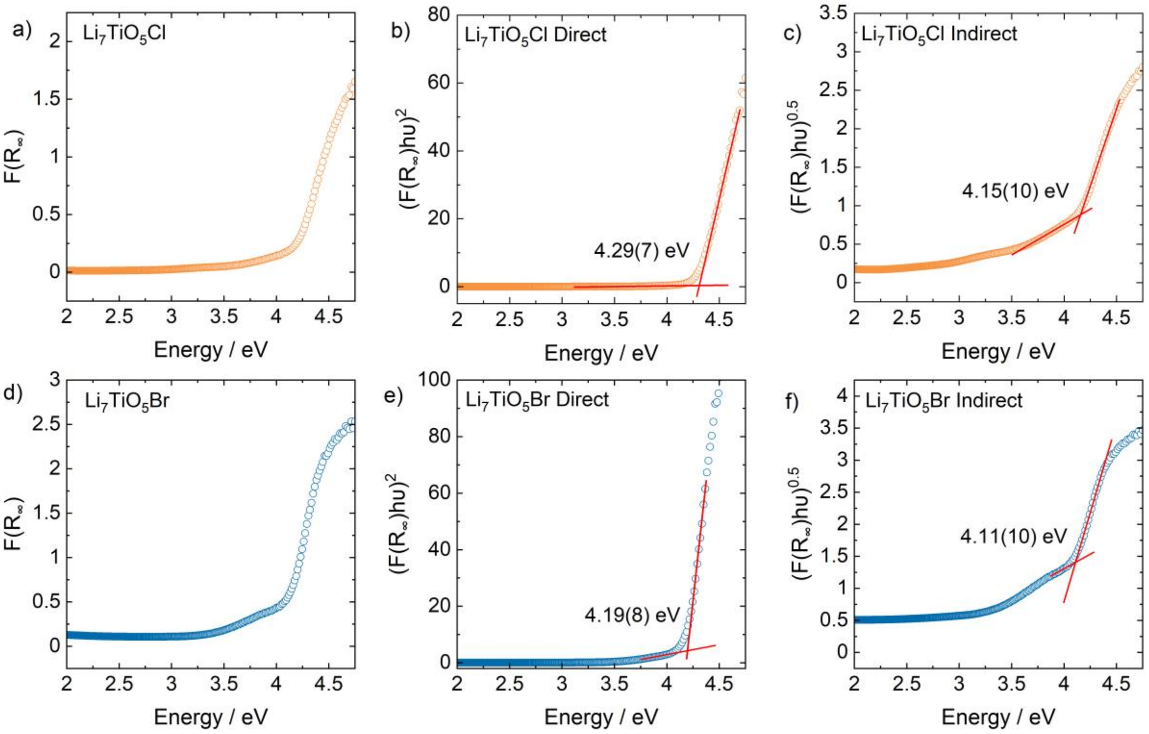

Figure S7: Diffuse reflectance data collected on a)-c) Li_7TiO_5Cl and d)-f) Li_7TiO_5Br powders. Tauc analysis of UV-vis spectrometry data enables extraction of direct band gaps of 4.29(7) eV and 4.19(8) eV for Li_7TiO_5Cl and Li_7TiO_5Br, respectively.

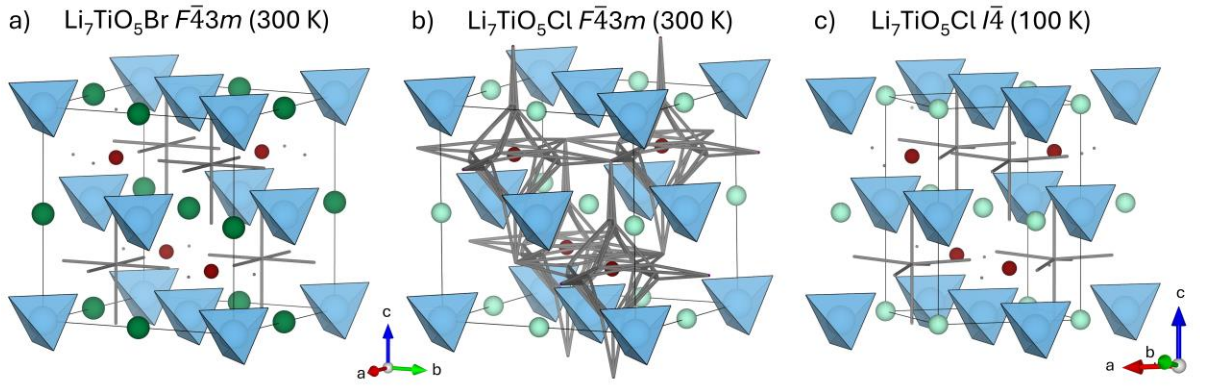

Figure S8: Structures of a) Li_7TiO_5Br ($F\bar{4}3m$) and b) Li_7TiO_5Cl ($F\bar{4}3m$) at 300 K, and c) Li_7TiO_5Cl ($I\bar{4}$) at 100 K. Neighbouring Li sites which are separated by <2.5 Å are connected by grey lines, which emphasises the importance of Li^+ site disorder to generate an extended 3D percolation network for Li^+ ion mobility in Li_7TiO_5Cl at 300 K. These extended networks are disrupted in the 300 K structure of Li_7TiO_5Br and 100 K structure of Li_7TiO_5Cl which exhibit Li^+ site ordering. Atom colours; Ti – blue, O – maroon, Li – grey, Br – green, Cl – eggshell blue. The O1 oxygen sites are omitted for clarity.

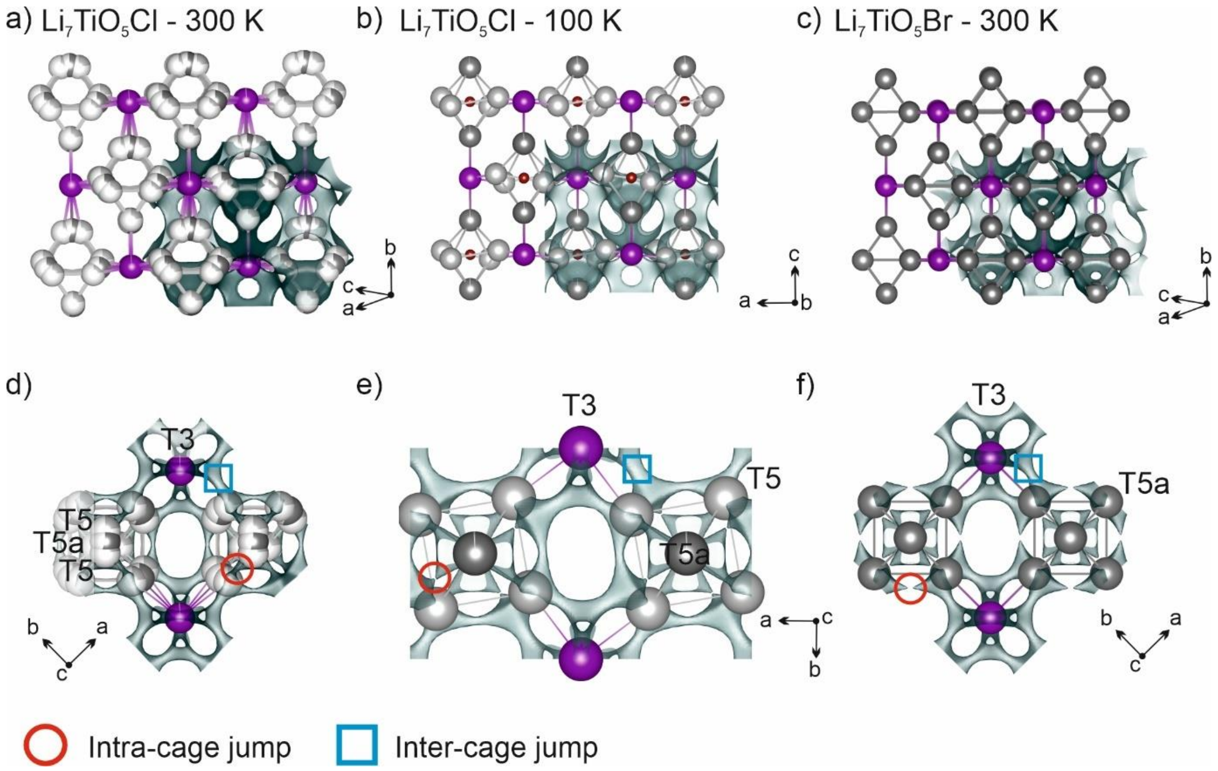

Figure S9: Bond valence site energy (BVSE) calculations for the a) Li-site disordered Li_7TiO_5Cl (300 K, $F\bar{4}3m$), b) Li-site ordered Li_7TiO_5Cl (100 K, $I\bar{4}$), and c) Li-site ordered Li_7TiO_5Br (300 K, $F\bar{4}3m$) structures with the BVSE landscape shown at an isosurface value of –1.00 eV. 3D diffusion pathways are evident in all three structures, but to a lesser extent in the Li site ordered structures. Panels d-f) show the BVSE landscape within and around the octahedral cages for all three structures shown at an isosurface value of –1.38 eV. Minimal difference is seen between inter-cage (T5–T3) jumps which are low energy and favourable for all structures, while the intra-cage (T5–T5) jumps are restricted in the Li-site ordered structures of e) Li_7TiO_5Cl (100 K, $I\bar{4}$) and f) Li_7TiO_5Br (300 K, $F\bar{4}3m$).

References

(1) Kong, S.-T.; Deiseroth, H.-J.; Reiner, C.; Gün, O.; Neumann, E.; Ritter, C.; Zahn, D. Lithium argyrodites with phosphorus and arsenic: order and disorder of lithium atoms, crystal chemistry, and phase transitions. *Chemistry* **2010**, *16* (7), 2198–2206. DOI: 10.1002/chem.200902470.

(2) Stokes, H. T.; Hatch, D. M. FINDSYM : program for identifying the space-group symmetry of a crystal. *J. Appl. Crystallogr.* **2005**, *38* (1), 237–238. DOI: 10.1107/S0021889804031528.
